# The Impact of SARS-CoV-2 on Sperm Cryostorage, Theoretical or Real Risk?

**DOI:** 10.3390/medicina57090946

**Published:** 2021-09-08

**Authors:** George Anifandis, Tyl H Taylor, Christina I Messini, Katerina Chatzimeletiou, Alexandros Daponte, Dimitrios Ioannou, Helen G Tempest

**Affiliations:** 1 Department of Obstetrics and Gynecology, School of Health Sciences, Faculty of Medicine, University of Thessaly, 41200 Larisa, Greece; pireaschristina@gmail.com (C.I.M.); daponte@med.uth.gr (A.D.); 2 Reproductive Endocrinology Associates of Charlotte, Charlotte, NC 28207, USA; tyltaylor@gmail.com; 3 1st Department of Obstetrics and Gynecology, Unit for Human Reproduction, School of Health Sciences, Faculty of Medicine, Aristotelian University of Thessaloniki, 56403 Thessaloniki, Greece; katerinachatzime@hotmail.com; 4 Department of Human and Molecular Genetics, Herbert Wertheim College of Medicine, Florida International University, Miami, FL 33199, USA; dioannou@fiu.edu (D.I.); htempest@fiu.edu (H.G.T.); 5 Biomolecular Sciences Institute, Florida International University, Miami, FL 33199, USA; 6 College of Medicine, Roseman University of Health Sciences, Las Vegas, NV 89135, USA

**Keywords:** sperm cryostorage, sperm cryopreservation, COVID-19, SARS-CoV-2-contamination

## Abstract

Cryopreservation of human gametes and embryos as well as human reproductive tissues has been characterized as an essential process and aspect of assisted reproductive technology (ART). Notably, sperm cryopreservation is a fundamental aspect of cryopreservation in oncological patients or patients undergoing gonadotoxic treatment. Given that there is a risk of contamination or cross-contamination, either theoretical or real, during the procedures of cryopreservation and cryostorage, both the European Society for Human Reproduction and Embryology (ESHRE) and the American Society for Reproductive Medicine (ASRM) have provided updated guidelines for preventing or reducing the contamination risk of sexually transmitted viruses. Given the ongoing and worldwide COVID-19 pandemic, there is considerable interest in what measures should be taken to mitigate SARS-CoV-2 contamination during cryopreservation and cryostorage of semen samples. The SARS-CoV-2 virus is the virus that causes COVID-19, and whose transmission and infection is mainly aerosol-mediated. Several ART professional societies, including ESHRE and ASRM have proposed measures to mitigate the spread of the SARS-CoV-2 virus. Whether the proposed safety directives are enough to mitigate the possible SARS-CoV-2-contamination of sperm samples during cryopreservation or whether the policies should be re-evaluated will be discussed in this review. Additionally, insights regarding the possible impact of COVID-19 vaccination on the safety of sperm cryopreservation will be discussed.

## 1. Introduction

Infertility affects millions of people worldwide and therefore, even during the COVID-19 pandemic, it remains an important public health issue. During the first months of the infection, many assisted reproduction technology (ART) professional societies published detailed COVID-19 guidance for the ART health community [1,2]. Initially, recommendations called for the suspension of non-essential ART, gamete and embryo cryopreservation services, diagnostic, and ART procedures [1,2]. These initial recommendations were established to reduce the risk of COVID-19 infections in patients and healthcare workers, and to reduce the load on healthcare systems that were already overburdened [3]. As the pandemic evolved and effective mitigation measures were implemented, European Society for Human Reproduction (ESHRE) and American Society for Reproductive Medicine (ASRM) provided updated recommendations regarding the re-initiation of critical, time-sensitive services whilst retaining the mitigating measures proposed initially [4,5]. In vitro fertilization (IVF) clinics have had to adapt to implement additional standard operating procedures as a precautionary measure against the highly contagious COVID-19 respiratory virus, thereby ensuring that good laboratory practices safeguard patients, personnel, gametes, and embryos [6,7,8].

Standard practice during ART is to assume any biological sample may be potentially infectious, and as such universal precautions are utilized. However, the precautions adopted are based on blood-borne viral infections, not respiratory-borne infections [7]. As more data on COVID-19 became available, concern was raised with the discovery of SARS-CoV-2 receptors in human gametes and embryos [9,10,11,12,13,14]. Data are mixed and some studies have failed to detect the virus in semen, either in acute or recovered symptomatic male patients [15,16,17,18,19,20] while others have demonstrated the presence of SARS-CoV-2 in the semen of affected males [21]. Conversely, Li and colleagues reported the presence of the virus in 15.8% of semen samples tested, in patients at the acute stage of infection and several patients who were recovering [21]. Furthermore, another study failed to detect the virus in semen samples in patients affected with SARS-CoV-2; however, they did report impaired semen parameters in COVID-19 male patients [16]. Both studies concluded that the virus might be present at undetectable levels, particularly in recovered patients [16,21]. SARS-CoV-2 has also been linked to infections of the testes and epididymis [13], which may lead to post-infectious perturbations in spermatogenesis. Furthermore, some COVID-19 patients are subjected to a hyper-inflammatory syndrome characterized by sustained fever and potential changes in the cytokine profile which may result in potential long-term effects on spermatogenesis [13].

A longer lasting impact of SARS-CoV-2 could be from the storage of potentially infectious material during the course of IVF. Viruses may retain their infectivity during cryopreservation and in storage at ultra-cool temperature [22]. Thus, sperm samples contaminated with SARS-CoV-2 could potentially cross contaminate other specimens during long term storage, making sperm cryopreservation a potentially risky ART procedure. Although there is no evidence that an aerosol-mediated virus retains infectivity at ultra-low temperatures, there is a “theoretical risk” of contamination [23]. Moreover, many factors such as the dilution of semen and the processing and removal of seminal fluid during the sperm cryopreservation process have demonstrated an impact on the presence of the virus in contaminated samples [24,25]. A recent study reported the absence of the virus in cryopreserved semen samples analyzed in 50 semen samples in January 2020 and 50 semen samples in April–May 2020 from the Hunan Province Human Sperm Bank (China) [26].

The present review aims to provide insights regarding the impact of SARS-CoV-2 on semen cryopreservation during the COVID-19 pandemic in conjunction with European and other directives upon the appearance of the virus as well as the virus variants as they emerge. Moreover, it will be comment on the “theoretical” and the “real” risk of contamination during the cryopreservation procedures which are considered as threats, as well as the appearance of the new SARS-CoV-2 variants which is also considered to be a threat during sperm cryopreservation.

## 2. Guidelines for Sperm Cryopreservation of Virus-Positive Samples, Including SARS-CoV-2

Sperm cryopreservation is standard practice within the ART community, allowing the utilizing of cryopreserved sperm during ART treatment and also preserving male fertility through long term sperm storage [27]. One of the concerns of long-term storage within liquid nitrogen is the detection of at least 28 viruses [28], including the SARS-CoV-2 virus [16,21], in semen samples. Although no cross-contamination of virus has occurred during ART (to these authors knowledge), ASRM, ESHRE, and CAP (College of American Pathologists) have provided specific guidelines to minimize cross-contamination of viruses, specifically sexual transmitted viruses [29,30,31]. Briefly, since semen samples may contain a variety of viruses at any detection level, several risk-reduction strategies are employed to minimize any potential risk of viral transmission during fertility treatments. One important measure to minimize any potential cross-contamination risk during sperm cryopreservation is the application of good laboratory practice. Standard laboratory practices published by ESHRE and ASRM involve repeated sperm washing steps, which lead to the significant dilution of any virus present in the sample. In addition to the washing steps, common semen preparation procedures (e.g., sperm swim up or density gradient centrifugation) also offer additional advantages. Firstly, these remove the seminal fluid which contains a significant amount of the viral load. Secondly, it separates the motile viable from the immotile non-viable spermatozoa leading to a smaller sperm volume that needs to be cryopreserved. Lastly, gradients and swim ups separate the sperm from other infectious cells within the ejaculate, thereby limiting the number of infectious agents within the sperm sample. An additional strategy that can be utilized is the use of closed vitrification devices or specific sealing techniques, which prevents the direct contact of the infected sample to liquid nitrogen. The use of closed systems in combination of sperm-washing methods has been demonstrated to decrease the viral load in cryopreserved samples [32]. Alternatively, the use of liquid nitrogen vapor as opposed to liquid nitrogen for long term storage of samples would significantly reduce the virus’s ability to migrate to other samples [33,34]. Although there is a lack of evidence of cross-contamination either using open or closed vitrification systems, good laboratory practices recommend that gametes and embryos from infected patients be stored in separate containers; thus, minimizing risk of cross-contamination (CAP checklist 2021). Additionally, a quarantine cryostorage tank is recommended for samples of unknown status until the specimen can be assessed for infectious potential.

Recently, the Canadian Fertility and Andrology Society published updated clinical practice guidelines [35] on the separation of specimens of unknown status. Their position was that the separation of specimens of unknown status is only administrative and not scientifically evidenced-based and therefore does not require separate cryostorage equipment. Therefore, samples of known positive virus status can be stored along with other samples [35]. Similar clinical practice guidelines for SARS-CoV-2 have been published by the respective Italian Society [36].

The European Centre for Disease Prevention and Control (ECDC) proposed precautionary measures in order to reduce the risk of contamination of human reproductive cells by viruses including SARS-CoV-2 [37]. Precautionary measures include the concept that all body fluids, including semen and follicular fluid, be treated as potentially contaminated [29] helps mitigate the risk of cross-contamination. This is of importance as viruses stored at ultra-low temperatures appear to retain their pathogenic properties [22] and therefore it is theoretically possible that cryostorage of an infected sample poses a potential threat even years later. Nevertheless, considering the series of events during cryopreservation the risk of cross-contamination appears to be negligible. The only examples that have been reported for cross-contamination were experimental studies using very high titers of infectious agents [38], which is in contrast to the cryopreservation setting of the IVF laboratory.

## 3. Theoretical vs. Real Risk

There are two types of risk, theoretical and real. Theoretical risk, as the name suggests, represents a perceived risk that could potentially occur, but has not yet. Conversely, real risk represents an actual “non-theoretical” that must be planned for? The possibility of SARS-CoV-2 cross-contamination during sperm cryopreservation is a “theoretical risk”. However, if the possibility of cross-contamination is perceived as a real risk, asymptomatic patients cryopreserving sperm at this time pose a potentially threat and challenge for ART programs. If we accept SARS-CoV-2 is likely to be similar in terms of resistance in liquid nitrogen as other viruses, in combination with the fact that SARS-CoV-2 may be detected in low titers in seminal fluid, SARS-CoV-2 could be classified as a potentially sexually transmitted disease, thus warranting the same protocols as cryopreserving samples from patients infected with a sexual transmitted disease. Given the current scientific evidence, the risk remains theoretical, and in our opinion, it is unlikely to pose any significant clinical cross-contamination risk. Additionally, there is accumulating evidence that SARS-CoV-2 is not detectable in semen of either recovered or infected patients and the fact that SARS-CoV-2 is aerosol-mediated suggest that cross-contamination of this virus is in our opinion a “theoretical risk”.

## 4. SARS-CoV-2 Variants and Vaccines

Theoretical risk constitutes a potential real threat with the appearance of variant strains of SARS-CoV-2. Viruses continuously change through mutation and SARS-CoV-2 is no exception. Numerous variants of the COVID-19 virus have been reported including variants that the Centers of Disease Control (CDC) have classified as Variants of Concern (VOC) as these variants appear to more transmissible or deadlier than the wild-type SARS-CoV-2 [39,40,41]. Thus, the contamination risk may need to be further evaluated with data regarding the current and future variants. Given that several VOCs have been shown to be more transmissible, it can be speculated that the presence of the virus in seminal fluid might be more detectable in comparison to the wild-type strain. With the increase in the presence and veracity of VOCs, it may be necessary to alter or address guidelines/recommendations. Therefore, the following question remains: will good practice in IVF laboratories provide enough protection to specimens from VOCs? One could argue that the supposed theoretical risk in this case is potentially increased given the limited knowledge regarding the detection levels of the variants in respective reproductive fluids. Some preliminary answers may be derived from vaccine efficacy data. Although a variety of vaccines have been developed aiming to mitigate the rate of COVID-19 infections, and so far, all provide some level of immunity to the variants. However, clinical trials of multiple vaccines in South Africa, where the B.1.351 VOC represents a significant proportion of circulating SARS-CoV-2, reported a lower vaccine efficacy when compared with trials in other countries where the B.1.351 was not dominant [42]. Therefore, new SARS-CoV-2 variants may need to be thoroughly investigated further and could potentially pose a real threat for cross-contamination. A practical solution remains to follow good laboratory practice, e.g., implementing the recommended washing steps to decrease the viral load to levels that are clinically insignificant. Another less practical and more costly option includes the use of closed vitrification devices.

Currently, there are a handful of SARS-CoV-2 vaccines authorized for use by both the European Medicines Agency and the Centers for Disease Control. These include mRNA vaccines (BNT162b2/Comirnaty, Pfizer-BioNTech and mRNA-1273 Moderna) and adenoviral vector-based vaccines such as the Johnson & Johnson’s Janssen COVID-19 vaccine. These vaccines target the highly conserved spike protein that is involved in receptor recognition, viral attachment, and entry into host cells [43]. The vaccines generate spike protein-specific antibodies, specifically anti-S IgG antibodies which have been shown to possess neutralizing activity against the first pandemic SARS-CoV-2 Wuhan HU-1 variant [44]. Although vaccination efforts are underway, emerging variants remain a significant cause for concern given that the antibodies generated through vaccination may no longer recognize the mutated virus. For example, the recent VOCs B.1.1.7 (United Kingdom) and B.1.351 (South Africa) have eight to ten amino acid changes or deletions in the spike protein that current vaccines target [44]. Although data are still emerging, initial studies suggest available vaccines still provide protection against current VOCs, but initial studies have reported that antibodies produced in response to vaccination neutralize the B.1.1.7 VOC, whereas neutralization of the B.1.351 VOC is reported to be reduced 8- to 13-fold [44]. As has been shown for the B.1.351 variant, it is possible that current vaccines may not provide the same degree of protection against current and future variants. Additionally, current vaccines may potentially be more or less effective at reducing transmission of the virus for different VOCs. Therefore, the reproductive processes may still remain in jeopardy, since we do not have any evidence concerning the detection of the virus in respective sperm samples or whether the variants will display any resistance during cryopreservation.

## 5. Conclusions

Although no case exists within the human IVF community of cross-contamination during the course of cryostorage, research has demonstrated that cross-contamination does pose a real risk [45,46]. If we accept that the SARS-CoV-2 virus can be detected in the semen and the virus is resistant to liquid nitrogen, as is the case with many other viruses, maybe additional specific measures should be followed to minimize the presence of the virus during sperm cryopreservation and any possible cross-contamination effect. Apart from the sperm washing steps, which are commonplace, a closed cryopreservation method could be applied to reduce the risk of cross-contamination further. This process involves the use of a closed “sealed” system, which prevents the sperm suspension from coming into direct contact with the liquid nitrogen and allows the cryopreservation of a relatively larger volume of semen (and subsequently more spermatozoa) [47,48]. The closed system for cryopreservation will all but guarantee that SARS-CoV-2 is not able to contaminate other specimens. Moreover, variants of this virus may have different cryopreservation characteristics, making each variant different in terms of cross-contamination ability. We still have much to learn about the function and the effect of SARS-CoV-2 and its variants on the various aspects of human reproduction [49]; we should be extra-cautious with the handling of gametes and cryopreservation [50].

## Data Availability

Not applicable.
